# Comparison of the AmpFire^®^ Multiplex HPV Assay to the Xpert^®^ HPV Assay for detection of human papillomavirus and cervical disease in women with human immunodeficiency virus: A pragmatic performance evaluation

**DOI:** 10.21203/rs.3.rs-2606441/v1

**Published:** 2023-03-02

**Authors:** Sikhulile Moyo, Doreen Ramogola-Masire, Natasha Moraka, Leabaneng Tawe, Farzad Noubary, Kesego Motsumi, Godiraone Manowe, Boitumelo Zuze, Botshelo Radibe, Faith TT Hungwe, Terence Mohammed, Comfort Maphorisa, Roger Shapiro, Simani Gaseitsiwe, Rebecca Luckett

**Affiliations:** Botswana Harvard Health Partnership; University of Botswana; Botswana Harvard Health Partnership; University of Botswana; Northeastern University; Botswana Harvard Health Partnership; Botswana Harvard Health Partnership; Botswana Harvard Health Partnership; Botswana Harvard Health Partnership; Royal Institute of Technology; Botswana Harvard Health Partnership; Botswana Harvard Health Partnership; Harvard T.H. Chan School of Public Health; Botswana Harvard Health Partnership; Beth Israel Deaconess Medical Center

**Keywords:** human papillomavirus (HPV), cervical screening, AmpFire, Xpert, human immunodeficiency virus (HIV), cervical precancer, cervical high-grade dysplasia, validation

## Abstract

**Background:**

Low- and middle-income countries (LMICs) account for nearly 85% of the global cervical cancer burden, yet have the least access to high-performance screening. International guidelines recommend human papillomavirus testing (HPV) as primary screening, yet implementation is inhibited by the cost of HPV testing.Atila AmpFire^®^ HPV Assay (AmpFire) is both affordable and easy to use, and offers individual genotyping. The objective of this study was to compare the performance of the AmpFire HPV assay to the Xpert^®^ HPV assay in detection of both HPV and clinically significant cervical disease.

**Methods:**

We utilized stored cervical specimens from a prospective cohortstudy of women living with human immunodeficiency virus (HIV) in Botswana conducted from May to July 2018. Positive and negative percent agreement was calculated for the AmpFire and Xpert assays, as was detection of high-grade cervical dysplasia.

**Results:**

63 stored cervical specimens haddetectable DNA after thawing and were included in the analysis. The positive percent agreement was 91.2% (95%CI: 76.3–98.1) and negative percent agreement was 79.3% (95% CI: 60.3–92.0). Six cases positive by AmpFire but negative by Xpert were HPV genotypes 35, 52 (n=2), 58, 68, and co-infection with HPV 45 and 68. Both Xpert and AmpFire assays detected HPV in all 10 samples of women who had high-grade cervical dysplasia.

**Conclusions:**

The AmpFire HPV assay demonstrated excellent analytic performance in both detection of HPV and clinically significant cervical disease. AmpFire HPV is a promising option to increase access to affordable, type-specific HPV screening for cervical cancer in LMICs.

## Background

Primary human papillomavirus (HPV) testing for HPV-associated cancer screening has become standard in high-income countries but is not widely available in low- and middle-income countries (LMICs).^1,2,3^ It is well established that HPV detection and genotyping is more sensitive than cervical cytology and histology for the diagnosis of cervical cancer.^4,5,6^ While LMICs account for nearly 85% of cervical cancer morbidity and mortality globally, they have the least access to high-performance screening tests.^7,8^

The greatest barrier to HPV screening in LMICs is the cost of the World Health Organization-prequalified HPV assays.^9,10^ A wide array of sensitive technologies used to genotype HPV including Aptima^®^ (Hologic Inc, USA), hybrid capture II (Qiagen, Australia) and Cobas^®^ 4800 (Roche Molecular Diagnostics, Switzerland) have been developed with the aim to reduce cost and increase throughput particularly in LMICs.^11,12,13^ The Xpert^®^ HPV Assay (Xpert, Cepheid, Sunnyvale, CA) has also sought to reduce cost and increase access to near-point-of-care HPV testing in LMICs.^14^ However, the price of the tests is still out of reach of many LMICs.

We recently reported that high-risk HPV testing combined with colposcopy has the highest sensitivity and positive predictive value for detecting high-grade cervical dysplasia in women living with HIV in Botswana.^15^ While Botswana has excellent laboratory infrastructure to perform laboratory-based HPV testing, implementation of HPV testing at the national level is hindered by the cost of HPV testing.^16^ In our research setting, high-risk HPV genotyping has been performed with the Xpert^®^ HPV Assay and the Abbott Real*Time*^Ò^ HPV assay, which both test for the same 14 high-risk HPV genotypes.^17^ While the performance of these tests in a research setting was satisfactory, their cost prohibits national implementation of primary HPV screening, despite demonstration that HPV-based screening is the most effective method to screen our population.

Atila AmpFire^®^ Multiplex HPV Assay (AmpFire) produced by Atila BioSystems (Mountain View, CA) is more affordable ($9 for 15 high-risk genotyping) than most available HPV assays and thus has the potential to increase access to HPV testing in LMICs.^18,19,20,21,22^ AmpFire is easier to use as it does not require extensive extraction and can be performed on any isothermal platform. The objective of this study was therefore to compare the AmpFire HPV assay to Xpert HPV assay using stored cervical samples, and to compare the performance of both tests for detecting clinically significant cervical disease.

## Methodology

### Study Population

We utilized 67 stored cervical swab specimens from a prospective cohort study of women living with HIV in Botswana conducted from May to July of 2018. The study investigated the performance of primary high-risk HPV testing followed by triage evaluation with cytology, visual inspection with acetic acid and colposcopy to diagnose pre-invasive cervical disease in women with living with HIV in Botswana. Histopathology outcomes were available for all HPV specimens which were positive by original Xpert testing.^15^

### Specimen collection

In the study, provider-collected samples were taken from the cervix for HPV testing and cervical cytology using a Cervex-brush^®^. HPV specimens were then placed in a PreservCyt^®^ transport medium and taken to the Botswana Harvard AIDS Institute Laboratory for HPV testing as previously described.^15^ All participants who tested positive for high-risk HPV returned for colposcopy (evaluation of the cervix using a microscope) and directed biopsy. Biopsy specimens were sent in formalin to the Botswana National Health Laboratory for processing and results were reported as benign, cervical intraepithelial neoplasia (CIN) 1, 2, 3 or invasive cervical cancer. Results were categorized according to clinical significance, where clinically significant pre-cancer or cancer included CIN 2, 3 or invasive cancer (CIN2+) and benign clinical disease included histopathological reports of benign and CIN1.

### HPV genotyping by Xpert^®^ HPV Assay

HPV genotyping was performed in the original study using the Xpert assay according to the instruction’s manual. The Xpert assay was used to qualitatively genotype the 14 high-risk HPV genotypes, including 16, 18, 31, 33, 35, 39, 45, 51, 52, 56, 58, 59, 66, and 68. The results were reported as HPV 16, HPV 18/45, and other high-risk HPV genotypes. The input volume of cervical cell suspension for this assay was 1mL per sample.

### HPV genotyping by AmpFire^®^ Multiplex High Risk HPV Assay

Stored specimens were thawed and prepared for assay processing in March 2021. One milliliter of each specimen was briefiy vortexed and transferred to a sterile 1.5mL eppendorf tube. The tubes were then centrifuged for 30 minutes at a maximum speed followed by a complete supernatant removal and subsequently 30μL of the lysis buffer was added and then thoroughly vortexed to resuspend the cell pellet. The whole content from each sample tube was transferred into a 0.2mL polymerase chain reaction (PCR) tube. Incubation of the PCR tubes was then performed at 95 °C for 10 minutes in a thermocycler.

Samples were then genotyped using the AmpFire by fluorescent detection according to the instruction manual (https://atilabiosystems.com/multiplex-high-risk-hpv-by-fluorescent-detection/). This assay detects 15 individual high-risk HPV genotypes in a single tube, including HPV 16, 18, 31, 33, 35, 39, 45, 51, 52, 53, 56, 58, 59, 66, and 68 (the same types as the Xpert HPV assay plus HPV 53). AmpFire uses the cyanine5 (CY5) fluorophore for HPV 16, the carboxyrhodamine (ROX) fluorophore for HPV18 and other high-risk HPV genotypes using the fluoresce in amidites (FAM) fluorophore, all combined with an internal control using the HEX fluorophore. The Real-Time PCR was programmed using Bioer Real-Time PCR system for an isothermal reaction setting of 60°C while taking fluorescence readings at the FAM/HEX/CY5/ROX channels once every minute for a total of 60 minutes. The thermocycling software system automatically reported the results of the cycle threshold values for each amplification curve in all fluorescence channels.

### Statistical Analysis

A convenience sample of approximately 70 stored specimens was planned based on available resources and feasibility. The statistical analysis of the relevant data was conducted using Microsoft Excel. HPV genotype agreement between the AmpFire and Xpert assays was reported as positive percent agreement (both results were positive), negative percent agreement (both results were negative), and discordant if the results of each assay were different. The 95% CIs of the positive and negative percent agreements were calculated. P-values were also calculated, and P < 0.05 was considered statistically significant. Comparisons to histopathology were descriptive.

## Results

67 stored cervical samples were available for validation. When Amp re was run on thawed specimens, 4 (6%) samples had invalid controls, leaving 63 samples with detectable DNA that was genotyped and included in the analysis. In the original study, Xpert detected HPV in 34 samples while 29 samples tested negative. AmpFire detected HPV in 37 of the 63 samples, classified into 13 of the 15 high-risk HPV genotypes detected by AmpFire (Table 1). Using Xpert as the standard, the overall agreement between AmpFire and Xpert was 85.7% (95% CI: 74.6–93.3). The positive percent agreement was 91.2% (95%CI: 76.3–98.1) and negative percent agreement was 79.3% (95% CI: 60.3–92.0).

Of the samples with discordant Xpert and AmpFire results, the three cases that were negative by AmpFire had been classified as “other high-risk HPV” by Xpert. The six cases that were positive by AmpFire but negative by Xpert were HPV genotypes 35, 52 (n=2), 58, 68, and co-infection with HPV 45 and 68. Additional notes on type-specific discordance are found in Table 2.

The results of the HPV assays were compared to the histopathologic diagnosis in the original study (Table 3). Among the women from whom the samples were collected, 10 had CIN2+ disease on histopathology. Both Xpert and AmpFire assays detected HPV in all 10 samples of women who had CIN2+, indicating that both assays would detect women requiring treatment to prevent progression to cervical cancer, which is the goal of HPV-based cervical screening. As expected, many women who were HPV positive by both AmpFire and Xpert assays had benign or no disease.

## Discussion

The AmpFire HPV assay demonstrated excellent analytic performance in detecting high-risk HPV genotypes, demonstrating 85.7% overall agreement with the Xpert assay. The AmpFire assay also performed equally well to Xpert HPV in identifying women with clinically significant cervical disease (CIN2+), thus meeting an essential requirement of a high-performance screening test. Our study also demonstrated the benefit of AmpFire in detecting individual HPV genotypes, as 13 of 15 high-risk HPV individual genotypes were detected in this small sample. Individual genotyping offers the possibility of HPV genotype restriction triage for positive HPV results, which is of increasing interest in cervical screening research.^23,24^ Finally, our study supports the performance of AmpFire in cervical screening in women with HIV, which is essential for increasing access to HPV-based screening in LMICs, which bear the greatest burden of HIV-attributable cervical cancer.^25^

These results show that an affordable and accurate HPV assay with individual HPV genotyping is within reach of the global market. Currently WHO recommends primary cervical screening with HPV assays with particular regulatory approvals.^26^ Our data support expediting the approval process for AmpFire in order to rapidly increase global access to affordable HPV screening.

The high rate of agreement between AmpFire and Xpert was most likely due to the similar real time fluorescence detection methodology and the same high-risk HPV genotypes detected by the two assays (aside from HPV 53 which is not included in the Xpert assay). Our finding is in keeping with prior studies evaluating AmpFire compared to WHO prequalified HPV tests in various specimen types. AmpFire successfully detected 15 high-risk HPV genotypes from cervical, vulvar and oropharynx tissue in paraffin embedded blocks with >95% agreement with Linear Array HPV DNA genotyping tests.^16,27^ Vaginal self-swabs had >95% agreement between AmpFire, both Roche Cobas^®^ 4800 HPV assay, and SeqHPV assay.^20^ Rates of concordant results between AmpFire and Roche linear array HPV on anal swabs was similarly high (90%).^17^ In a population of women living with HIV, vaginal self-swabs were found to have 89% positive agreement between AmpFire and Xpert, with AmpFire being slightly more likely to diagnose HPV than Xpert.^28^ Beyond agreement in HPV results, our findings demonstrate the accuracy of AmpFire in identifying women with CIN2+, data which few other studies have con rmed.^20,25^

It is not clear what accounts for the differences in detection of HPV between assays. The use of different materials for the detection process could be a factor to consider. AmpFire produces cell lysates using the kit extraction lysis buffer, whereas Xpert performs cell lysis and purification within the cartridge, ensuring high quality HPV DNA before PCR amplification. Another possible factor is the open reading frames targeted by the Xpert assay which are E6/E7 whereas the AmpFire assay targets E1 and/or L1. Process monitoring to further optimize performance is warranted.

The AmpFire assay is well-suited for HPV detection in most resource limited settings aiming to implement HPV testing. The ability to detect HPV DNA in samples that had been frozen for three years in a practical setting is reassuring; samples can be collected, stored, and tested at a later time when stored in the correct conditions for prolonged periods. Data from other studies support the stability of HPV DNA on dry swabs across unregulated temperatures for at least a month which increases the feasibility of conducting population-based screening with decentralized self-collected vaginal HPV samples that could be stored in variable conditions until transport and testing are possible in a referral laboratory.^29,30^ Other attractive features of the AmpFire HPV assay include ease of use in the laboratory setting, particularly with minor sample preparation for lysis with no need for extensive extraction. There is a short hands-on time to process specimens and it requires only a small sample volume. It uses isothermal amplification, which can be run on any Real-Time PCR or isothermal platform and has a rapid turnaround time to results. AmpFire requires a laboratory infrastructure at present; however, it approximates the requirements for speed and ease of use for point-of-care applications, including a rapid preparation and run-time, and has been used in a same day test-and-treat project.^31^ With further optimizations and modifications, AmpFire has the potential to integrate clinic-based HPV testing into same-day test-and-treat programs in LMICs.

This study has several limitations. Our study used a small sample of stored specimens due to feasibility constraints. Additionally, we only have histopathology data on women with positive HPV results, limiting our ability to evaluate the sensitivity and specificity of both assays in detecting CIN2+ in the entire sampled population. Another limitation is the lack of gold standard used as a comparator. However, the Xpert assay is WHO pre-qualified, and in validation of other infectious disease tests it has generally been acceptable to evaluate the interchangeability of a test with a clinically utilized comparator without altering diagnostic accuracy in cases where a gold standard is not available.^32^ Given the need to expand HPV testing in LMICs, and the affordability of the AmpFire assay relative to other tests on the market, we took this practical approach to our validation in order to expedite its utilization. The high level of agreement observed between the assays is reassuring as agreement should theoretically decrease when comparing against an imperfect reference standard.^33^

A larger study with a true gold standard (histopathology for all specimens) is necessary to evaluate the performance of AmpFire in the detection of high-grade cervical dysplasia. This study is currently underway by our research group in Botswana and will allow validation of the accuracy of AmpFire in diagnosing CIN2+ in 3000 women. The study will also include histopathologic assessment of a proportion of the women who test negative with AmpFire, such that test performance parameters (sensitivity, specificity, positive predictive value, negative predictive value) can be evaluated.

## Conclusions

The AmpFire HPV assay demonstrated excellent performance compared to a clinically utilized alternative and is a promising option to increase access to affordable, type-specific HPV screening for cervical cancer in LMICs. Because of its simplicity, low cost, and ease of use, AmpFire can be easily implemented in facilities that currently have no HPV testing. Expedited approval is essential to rapidly scale-up affordable HPV primary screening for cervical cancer.

## Figures and Tables

**Table 1:** Agreement between AmpFire and Xpert HPV assays

		Xpert HPV		
		POS	NEG	Total
Atila AmpFire	POS	31	6	37
	NEG	3	23	26
	TOTAL	34	29	63

**Table 2:** Distribution of the HPV type by assay

Xpert^1^ HPV (n=34)		AmpFire^2^ HPV (n=37)		Comments regarding discordance
HPV genotype	Frequency^3^	HPV genotype	Frequency^3^	
HPV 16 only	4	HPV 16	3	1 Xpert HPV16 positive was AmpFire HPV 18,59 positive
HPV 18/45	8	HPV 18	5	1 Xpert 18/45 positive was AmpFire HPV33 positive only 1 Xpert 18/45 positive was AmpFire 18+45 positive 1 Xpert 16+other positive was AmpFire 16+18+35 positive 1 Xpert other high-risk positive AmpFire HPV18+35 +68 positive
		HPV 45	6	1 Xpert negative was AmpFire HPV45+68 positive
Other HPV	26	HPV 31	2	3 Xpert other high-risk positive were AmpFire negative
		HPV 33	7	
		HPV 35	4	1 Xpert negative was AmpFire positive
		HPV 39	2	
		HPV 51	0	
		HPV 52	10	2 Xpert negative were AmpFire positive
		HPV 53	0	
		HPV 56	1	
		HPV 58	2	1 Xpert negative was AmpFire positive
		HPV 59	3	
		HPV 66	0	
		HPV 68	8	1 Xpert negative was AmpFire positive

1 Xpert^®^ HPV Assay (Xpert, Cepheid, Sunnyvale, CA),

2 Atila AmpFire^®^ Multiplex HPV Assay (AmpFire) produced by Atila BioSystems (Mountain View, CA

3 Some co-infections included so individual samples may be represented multiple times by the different HPV genotypes detected.

**Table 3.** Comparison of AmpFire^®^ and Xpert^®^ HPV in predicting histopathology among 63 validated samples

		Atila AmpFire^®^		Xpert^®^ HPV	
		POS	NEG	POS	NEG
Pathology	CIN2+ (disease)	10	0	10	0
	<=CIN1 (no disease)	27	26	24	29

## Data Availability

The datasets used for the current study are available from the corresponding author on reasonable request.

## Abbreviations

Atila: Atila AmpFire^®^ Multiplex HPV Assay (Atila BioSystems, Mountain View, CA)
CIN: cervical intraepithelial neoplasia
CY5: cyanine5
FAM: fluoresce in amidites
HIV: human immunodeficiency virus
HPV: human papillomavirus testing
LMICs: Low- and middle-income countries
PCR: polymerase chain reaction
ROX: carboxyrhodamine
Xpert: Xpert^®^ HPV Assay (Xpert, Cepheid, Sunnyvale, CA)
